# Dispersion and Disparity: Bibliometric and Visualized Analysis of Research on Climate Change Science Communication

**DOI:** 10.3390/ijerph192315766

**Published:** 2022-11-26

**Authors:** Denghang Chen, Yanlong Guo, Chenyang Wang, Yinrui Xu, Han Zhang

**Affiliations:** 1 Department of Science and Technology Communication, University of Science and Technology of China, Hefei 203106, China; 2 Research Center for Science Communication, Chinese Academy of Sciences, Hefei 203106, China; 3 Social Innovation Design Research Centre, Anhui University, Hefei 203106, China; 4 College of Environmental Science and Engineering, Ocean University of China, Qingdao 266000, China

**Keywords:** climate change, science communication, knowledge graph, bibliometrics, developing countries

## Abstract

Research on climate change science communication began in the 1980s and is showing continued vitality and a wider interest at present. In order to track the development of global research on the communication of climate change hot topics and frontier progress since the 21st century, methods such as bibliometrics and co-word network analysis were used to analyze the publication of research papers in this field, and a total of 1175 valid papers published in 2000–2021 in the WOS core database were counted. Different dimensions such as temporal trend, spatial distribution, and author collaboration network were analyzed. The results show that, (1) climate change communication research has become a relatively independent research field and has entered a rapid development stage, and this field still has a broad research prospect in the new understanding of climate change and new international context. (2) At present, research in this field is still dominated by developed countries, but developing countries are actively building their unique climate communication discourse. (3) Public understanding and media information presentation have been hot topics in climate communication research in recent years. In the context of changing international situations and the development of global epidemics and new climate policies, changes in national actions will likely lead to new research topics and dialogues. Research shows that climate change science communication research is increasingly showing a trend of decentralization and differentiation.

## 1. Introduction

Since Swedish researcher and Nobel laureate Svante Arrhenius published his report on human-caused global warming in 1896 [1], climate change has entered the scope of scientific research. Climate change was defined as a purely environmental issue in the early perceptions of scholars worldwide, and before the 1970s, meteorological scientists believed that climate characteristics could be described in units of 30-year average climate conditions. It was not until the 1990s that the dynamic and nonindependent nature of the climate change system gradually gained academic recognition, and the abruptness of climate change reduced the time unit for characterizing climate to a decade or even less [2,3].

The phenomenon of climate communication has emerged almost simultaneously with the issue of climate change, and climate communication has played an important role in the response to climate change. Thus, in early climate communication practices, scientific findings, extreme weather events, political summits, and regular reports issued by the Intergovernmental Panel on Climate Change (IPCC) were given more coverage [4]. As the issue of climate change has received increasing social attention, research on climate communication has been on the rise. Climate communication researchers generally believe that anthropogenic climate communication research originated in the 1980s, with the United States and the United Kingdom being the earliest countries to study climate change communication. Researchers from a variety of disciplines, including environmental science, psychology, political science, and communication studies, have conducted a wealth of research as early as the late 20th century to early 21st century [5].

Previously, there have been attempts to summarize and analyze the progress of climate change communication research, such as Moser’s evaluation of the essential advances and rising developments in research of climate change communication during the period from 2000 to 2010 [6]. Asmi et al. focused on the knowledge structure and development frontiers in the same research area [7]. In addition, some researchers have tried to narrow the boundaries of their studies, for example by analyzing the profile of climate communication research in China and Western countries, or by targeting specific journals for their studies [8,9,10,11].

However, previous studies either failed to take note of the latest research or were based only on selective samples. Climate communication is currently generating wider interest at present, with the release of the IPCC Sixth Assessment Report, which describes the newest understanding of natural science and the situation of major regional climate change worldwide. The 26th Conference of the Parties to the United Nations Framework Convention on Climate Change concluded with the signing of the Glasgow Climate Pact by representatives of nearly 200 countries, emphasizing the importance of science in policy-making to address climate change. The 2021 Nobel Prize in Physics was awarded to climatologists Manabe and Hasselmann for their outstanding contributions to how humans affect the climate [12,13]. At the same time, the COVID-19 pandemic and the outbreak of war triggered a change in climate policy in several European countries, which drew a lot of attention from environmentalists and was accompanied by extreme behavior, and the politics and sustainability of climate communication became the subject of much discussion.

Therefore, we would like to visualize and analyze the global literature on the topic of science communication of climate change since 2000 through bibliometric analysis under the purpose of addressing the following three key research questions (RQ):

RQ1: How far has the current scientific research on climate communication progressed worldwide?

RQ2: Who, and which institutions and countries constitute the major groups in climate communication research?

RQ3: What are the hot topics and frontiers in climate communication research?

With the three research questions mentioned above as the main line, the remainder of this article is organized as follows. Section 2 describes the methodology and data sources used in this study, as well as the data processing process. Section 3 discusses the historical trends in the publication of climate communication research papers, and the fourth section analyzes the geographical variability in research in this field, the main contributors and the collaborative networks among them. The fifth section identifies hot topics of research, changes in research themes, and frontiers. Finally, Section 6 provides answers to the three research questions and indicates future research directions.

## 2. Data Sources and Research Methodology

### 2.1. Data Sources and Collection Procedure

The literature source of this paper is the Web of Science core collection database, from which we collected data on April 5, 2022. To ensure that first-hand data are comprehensive, have a high degree of interpretation, and as many samples as possible are obtained, topic research rather than advanced research was used. The literature was searched with the formula of “TS = science communication of climate change”. The first search conducted produced 1933 articles, and then the literature was filtered according to the time distribution of the number of articles, and the time span was set from 1 January 2000 to 31 December 2021. Considering the quality of literature and cross-sectional comparison factors, the sample literature was subjected to data cleaning work, excluding review papers, conference proceedings papers, book reviews, editorial materials and unrelated disciplinary literature, and a total of 1559 literature were retrieved, and 1175 valid literature were obtained after further screening based on research topics and literature content.

**Figure 1:**
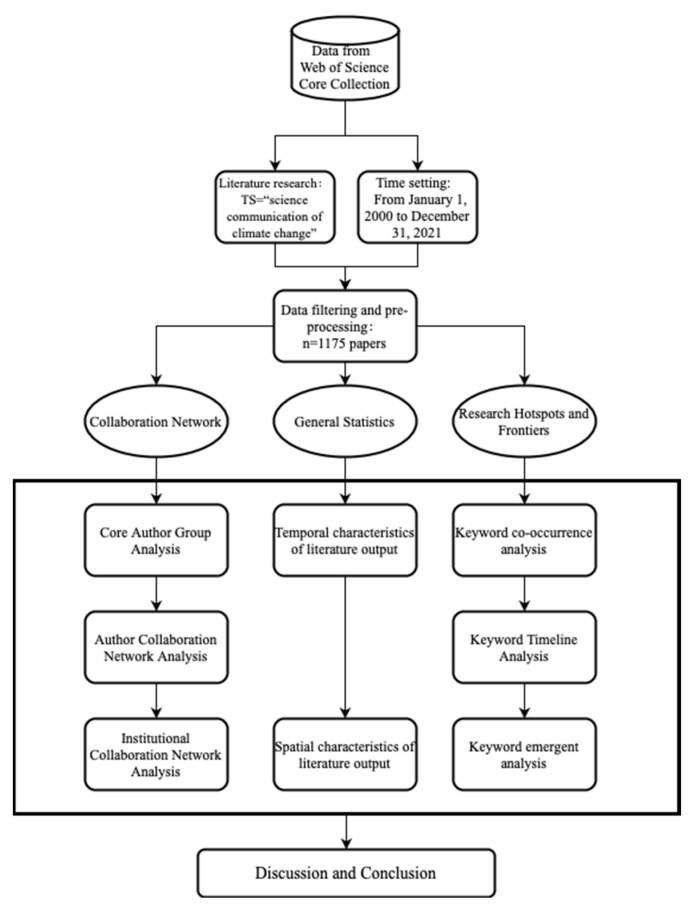
The integrated analysis framework.

In the analysis of collaborative networks and general statistics, we set the node type as author, institution, and country, respectively, and used the cosine algorithm as the calculation of linkage strength. In the analysis of research hotspots and frontiers, keywords were selected as node type, noun terms were extracted from the original keywords of authors and supplementary keywords of the dataset to name the clusters, and the log-likelihood ratio (LLR) was used for the extraction of cluster labels. The time slice in all operation steps was set to 1 year, and the threshold (k-value) was set to 25. The specific research route of this study is shown in Figure 1.

### 2.2. Research Methodology and Tools

Bibliography can be traced back to 1917 [14] when Hume first introduced the concept of “Statistical Bibliography” in 1923, and by extending the object of study from academic journals to all available literature, bibliographers developed bibliography into bibliometrics [15,16].

In order to achieve a bibliometric analysis of the literature, in addition to statistical analysis of the time and country related to the literature using descriptive statistics, keyword co-occurrence analysis and author collaboration network analysis were used to analyze the collaboration of hot topics and publications published in this research area and to track important research areas and developments.

This study uses CiteSpace analysis software in the field of bibliometrics. Based on a path-finding network algorithm (pathfinder), as well as co-citation analysis theory (co-citation), CiteSpace is capable of identifying the research progress, new trends and new developments in a certain field in scientific development and presenting them in a visual form for users’ regular discovery and decision support [17]. It applies to multivariate, time-phased, dynamic and complex networks and is currently used in more than 10 countries and regions, and is also a commonly used tool in the field of scientometrics [18,19,20,21].

## 3. Temporal Distribution and Trends of Publications

**Figure 2:**
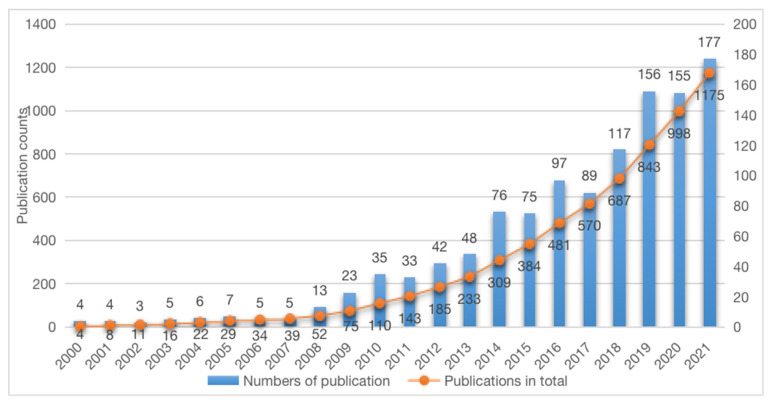
Chronological distribution of the number of climate change science communication research papers from 2000 to 2021.

The number of annual publications can reflect the level of development of a research field to determine its research development [22]. Based on the historical literature included in the Web of Science (Figure 2), it can be found that the topic of climate change science communication did not receive much attention from academics until 2007. Since 2008, research on this area has entered a phase of rapid growth, reaching a peak in 2021.

In terms of the time scale of the number of publications, climate change communication research can be divided into three stages: the preliminary stage, the stable growth stage, and the rapid development stage. In the preliminary phase (2000–2007), only 39 articles appeared in eight years, which represents 3.3% of all the literature. During the five years of the steady growth phase (2008–2013), 194 articles were published, almost five times the total number of articles in the first phase, accounting for 16.5% of the total literature. This indicates that researchers during this period began to show interest in climate change communication by rapidly publishing research results and occupying priority in scientific discovery. In the third period (2014–2021), the number of publications occupies 80.2% of the total literature, with an average of 117.8 publications per year. This indicates that climate change communication research has formed an independent research direction and become a more popular research area.

The increase in research literature from 2008 onward is mainly attributed to the following two factors: (1) the advancement of natural science, which has revealed the impact of human activities on climate change and the deterioration of the human living environment; sensing the environmental crisis of human existence has led social scientists to explore ways to promote environment-friendly behavior [23]. (2) The social construction of science. More and more countries and nonprofit organizations have become aware of the relationship between climate change and human life and have promoted negotiation and collaboration between countries, and the high level of international and public concern has triggered researchers to pay attention to the communication of climate change [8]. The Copenhagen Conference held in 2009 is a typical example. After this conference, research outputs related to climate change communication show a significant increase and the research in this field began to enter a new era of rapid development, raising the attention of governments, the public, enterprises, and other diversified social actors so that climate change became the frontier of research.

According to Price’s law, many indicators in science grow according to an exponential law. In other words, the growth rate of various scientific indicators is proportional to the existing values of the indicators. The exponential curve estimation analysis using SPSS 25.0 software yielded the exponential function of the curve as:

y = 24.957e^0.027x^ (1)

The results showed that R^2^ = 0.791, *p* < 0.001, which is consistent with Price’s law, indicating that climate change communication research is still in a rapid development phase at this time.

## 4. Spatial Variation and Cooperative Network Analysis

### 4.1. Spatial Distribution of Studies

**Figure 3:**
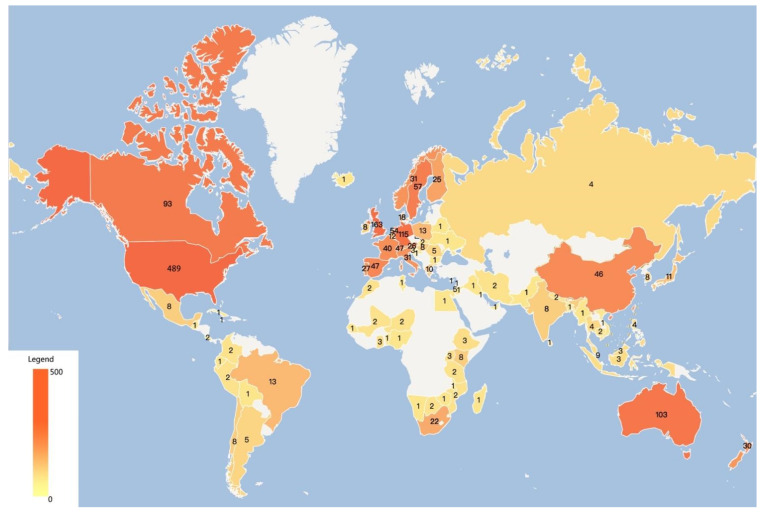
Geographical distribution of literature.

Statistics on the countries in which the literature is published can reflect the importance of the country and its influence in a particular field of research [24]. When different authors, institutions and countries appear in the same paper, CiteSpace assumes that a collaborative relationship was created in the writing of the paper. Therefore, all countries where the authors were located are included in the statistics. If multiple authors of a paper were from the same country, this country was counted only once. Figure 3 plots the distribution of literature output by country in the scientific research of climate change communication, and research in this domain has been mainly concentrated in Europe and North America, which echoes the earlier qualitative studies [25]. Among them, the U.S. is not only the first country to study climate communication issues, but also the region with, by far, the highest number of publications. Although the UK, Germany, Australia and Canada are among the top 5 countries with some research accumulation with the U.S., there is still a large gap between the four countries and the U.S. The number of studies in Asia, South America and Africa are generally low, and scholars from countries other than the United States have focused on this area and conducted research relatively late, mainly between 2008 and 2010.

Among the top 20 countries to which the authors of the papers belonged (Table 1), all of them were developed countries except China and South Africa. This indicates that there is a correlation between the level of economic development and climate change communication research, developed countries are more concerned with scientific communication research on climate change. On one hand, developed countries generally have stronger academic research capacity and academic innovation than developing countries, and are also more involved in the theoretical exploration of climate communication at an earlier stage. On the other hand, for developing countries, economic development and poverty eradication are the primary goals of these countries in the industrialization process, so in the practice of scientific research, research related to climate change communication was conducted later than in developed countries. However, as the response to climate change becomes a global governance activity, developing countries are also actively involved in this action and related research, and their research focused on building a framework for climate communication discourse in developing countries, for example, China as the representative of developing countries, hoping to fight for more space for survival and development for developing countries in international climate negotiations and to assume common but differentiated responsibilities [26,27,28,29]. 

### 4.2. Analysis of Core Authors

Authors are the foundation of the research field and the source of innovation, and core authors represent a cluster of researchers who have contributed significantly to the improvement of a certain research field. Table 2 shows the top 20 scholars in the field of climate communication research in terms of the number of publications. The well-known bibliometrician Price proposed that authors with m or more publications are outstanding scientists, i.e., core authors, calculated by the formula [30]:

m = 0.749 (n_max_) ^1/2^ (2)

In the above formula, n_max_ is the number of articles published by the highest-producing author. Anthony Leiserowitz is the most prolific author in the area of climate change communication, with a cumulative total of nine publications, from which the value of m is calculated to be 2.247. After rounding off, all authors with a number of publications greater than or equal to 3 can be considered core authors in the field of climate communication, and there are 16 of them.

From the analysis of the statistical results, it is clear that the total number of publications by core authors of climate communication research between 2000 and 2021 is 67, accounting for only 5.7% of the total number of publications, which is far from the requirement that half of the papers in this field are written by core authors as stated by Price. Therefore, there is currently no large and stable core group of authors in the worldwide climate change science communication research community. In total, 14 scholars have single articles with 20 or more citations, and Kahan’s study, published in 2012 and cited in 38 papers to date, suggests that public disinterest and disagreement about climate change stems from a unique conflict of interest that includes individual and collective interests [31]. Another equally highly cited article comes from Moser, who in 2016 reviewed important advances and emerging trends in climate communication and received extensive attention [6]. Unlike selective literature analysis, Cook and his team have received the same peer attention by analyzing the evolution of the scientific consensus on anthropogenic global warming (AGW) in the scientific literature through a bibliometric approach [32]. 

### 4.3. Collaborative Network of Authors

**Figure 4:**
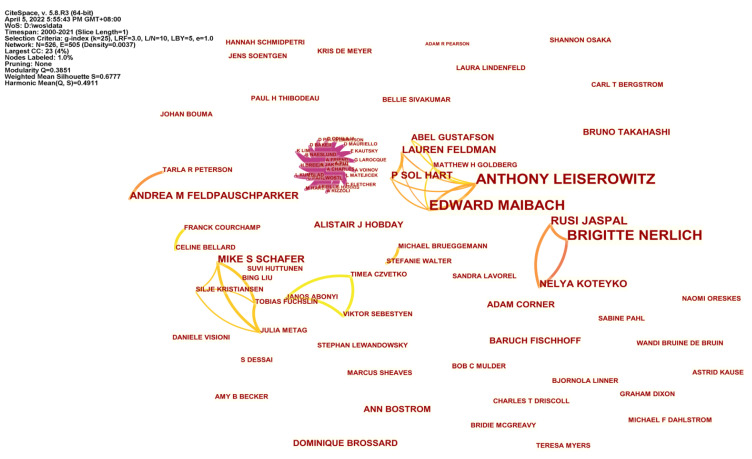
The collaborative network of authors in the field of climate communication research.

The analysis and visual presentation of the network structure among authors can clearly demonstrate the cooperative relationships among scholars and institutions, and provide a new perspective for the evaluation of academic impact in this field. From Figure 4, we can see that there are 526 authors, and the number of collaborations is 505. The collaboration density (D) is presented by the ratio of the actual number of relationships (m) of a node to the theoretical maximum number of relationships in a network with n nodes, which is 0.0037. The calculation formula is [33]:

D = 2m/[n(n − 1)](3)

In the case of low overall collaboration density but more collaborative relationships, it indicates that a few articles contain a large number of the author–author collaborations, which also indicates that most author collaborations in the research of climate change communication are dominated by small-scale collaborations and individual studies. Thus, the collaboration among author groups is expected to be further strengthened.

Through our analysis, we found that a stable, long-term collaborative network has formed among the core authors. For example, Leiserowitz, Gustafson and Goldberg experimentally test how perceptions of natural gas vary depending on what it is called; they also analyzed how targeted advertising shifts Republican views on climate change [34]. Leiserowitz and Maibach also investigated public perceptions of the human health risks of climate communication in the United States, Canada, and Malta [35].

Although the worldwide collaboration of researchers working on the topic of climate change science communication is fragmented, the field of climate communication has produced several stable collaborative networks (Figure 4), namely Tarla Peterson and Andrea Feldpausch-Parker, Franck Courchamp and Celine Bellard, and Michael Brueggemann and Stefanie Walter. The node size is proportional to the number of articles posted by the authors, and the connections between nodes represent how closely the authors collaborate. Michael and Stefanie have co-authored a total of six articles, four of which are about group perceptions of climate change and scientists’ media-use behavior in climate change. Jaspal has established a collaboration with two other researchers, Nerlich and Koteyko, and they examined the rhetorical aspects of social debates about climate change in the Daily Mail’s reader comments [36]. In addition, they collaborated to analyze remarks posted on the British tabloid internet site at two different points in time earlier and later than the East Anglia controversy [37]. Sebestyen, Czvetko and Abonyi highlight the applicability of data and models in complex climate change research issues [38].

### 4.4. Institutions and Cooperation Network 

Collaborative network mapping was used to analyze the main contributing institutions and the relationship between them. The findings indicate that the relatively influential institutions in the climate change communication research field are concentrated in the UK (Table 3). The U.S. and other developed countries, with George Mason University, Cornell University, Oxford University, and Cardiff University, started to focus on climate communication issues before 2010, having accumulated rich research results in this research area. It is noteworthy that the Chinese Academy of Sciences, which first began to emerge in the research results on climate change science communication in 2015, has accumulated 17 papers so far, ranking sixth, with rapid growth in results. The above mentioned institutions, together with the University of British Columbia, the University of Michigan, and Yale University, have an extremely important position in the research field of climate change science communication, and cooperate more closely with other research institutions, assuming an important linkage role.

**Figure 5:**
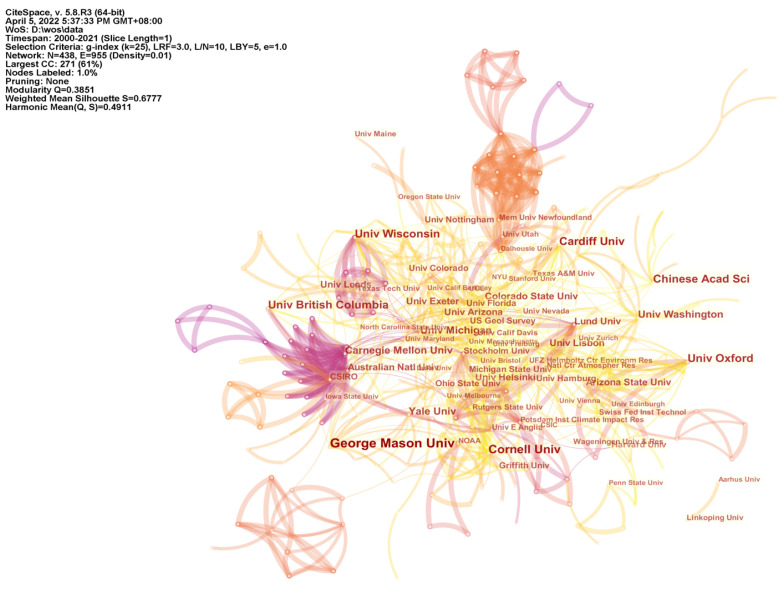
Institutional cooperation network.

The lines emitted from a node indicate the collaboration between nodes, and larger nodes and more lines mean a closer research collaboration. By setting the node type to institution, we calculated the number of emissions and cooperation networks of research institutions (Figure 5). The analysis found that 955 connecting lines were formed between 438 institutions, with a cooperation density of 0.1. This indicates that there is closer cooperation between different scientific research institutions. George Mason University has accumulated some theoretical results in differentiated audience research, including elites, cooperative extension professionals, students, and adolescents [39,40,41,42]. Cornell University tends to look at ways to more effectively promote the practice of climate change science communication by developing a climate change information system or new approach for evaluating climate change communication. What is more, they examined the link between coffee consumption and climate change communication and found that the coffee consumption experience can be regarded as a practical tool for public participation in climate change [43,44]. The Chinese Academy of Sciences uses bibliometrics to study research trends in environmental communication and considers the lack of international cooperation as a major shortcoming in the field of environmental communication research [45]. 

## 5. Research Hotspots and Research Frontiers in the Field of Climate Change Communication

### 5.1. Keyword Co-Occurrence Analysis

**Figure 6:**
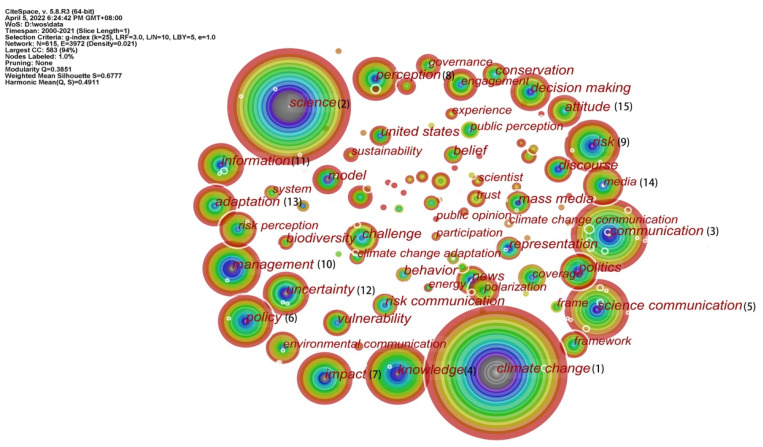
Co-occurrence of keywords.

The frequency of co-occurrence refers to the number of times a node appears in the network, and the frequency of co-occurrence directly reflects the research hotspot [46]. The size of the tree ring reflects the frequency of keywords. The co-occurrence analysis of keywords (Figure 6) revealed that “climate change”, ”science”, “communication “, “science communication”, “knowledge” and “policy” are high-frequency terms from 2000 to 2021. This reveals that knowledge about climate change, knowledge diffusion and climate change-related policies were the hot topics of research during this period. From Table 4, we can see that “climate change” was the earliest and the most frequent term, with a frequency of 645. Science and communication were the second and third keywords, respectively, and both appeared for the first time in 2005. There are 8 keywords with frequencies greater than 100, and 15 words with frequencies above 60. In addition to climate change and science communication always being the core keywords in this field, how to reduce social risks and uncertainties, and public attitudes and behaviors have also become popular topics of interest for researchers.

### 5.2. Analysis of Main Topics

Keyword clustering can reflect the research clusters formed in a subject area by clustering closely related keywords into one category. The data showed that the keyword clustering network density for climate change propagation in keyword clustering and timeline analysis was 0.021, Silhouette values characterizing keyword homogeneity were 0.6777, and Modularity values were greater than 0.3, which indicated high homogeneity of the mapping network and reasonable clustering results.

**Figure 7:**
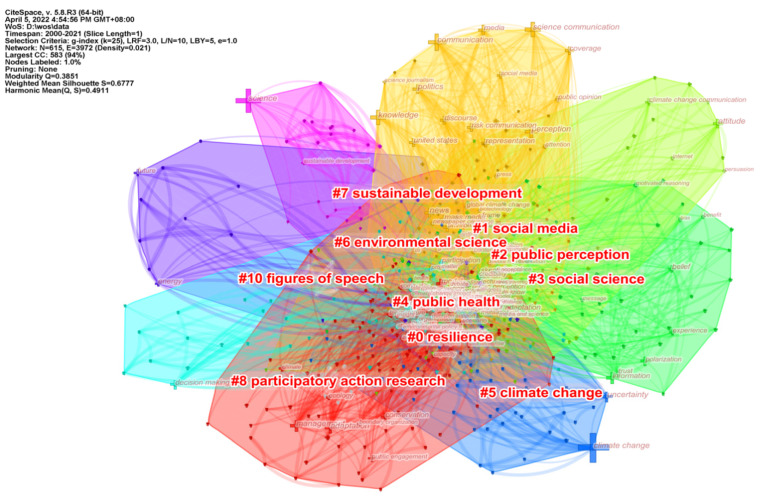
The keyword cluster map for the field of climate change science communication.

Literature in the same cluster is placed in the same color block, and the more literature in a cluster means that the more important the cluster area is. The closely related keywords were grouped into 10 major clusters (Figure 7), and the clusters were divided into three main categories to provide a clearer picture of the main research themes in the field of climate change communication.

(1) Various topics based on climate change in the natural sciences (cluster#5, cluster#6, and cluster#9). Due to the impact of climate change on all aspects of human life, scholars have generated a variety of research topics around climate change in their respective research fields, but in general, they hope to promote the understanding of climate change in the scientific community and the public through their respective research. For example, in the field of science education, Bell Wayne and others from Washington College use real environmental data lessons to improve students’ understanding of basic science concepts. In the area of the Internet, Salam, A. has attempted to build a framework for the Internet of Things, hoping to enhance the understanding of regional climate through the use of technological solutions [47,48]. 

(2) Climate Change Science Communication (cluster#1, cluster#2, cluster#3, and cluster#10). This is central to the field of climate communication, with environmental communication, social science, and rhetoric as keywords in this category, and researchers have worked to identify how messages are communicated and the role of media [49,50]. They also investigate how social science methods and practices can raise awareness of climate change policy, economics and culture among student groups, and engage them in thinking about the topic of climate change and science communication [51,52]. 

(3) Public Policy (cluster#4, cluster#7, and cluster#8). Because climate change poses a multifaceted and complex threat to human health and well-being, researchers want to alert and examine how to communicate this threat and health risks to the public [53].For instance, exploring possible pathways for changing dietary habits and health behaviors to reduce carbon emissions through programmed behaviors to slow the rate of climate degradation [54,55]. Others compared policies to address climate change in different countries around the globe and explored and identified key government departments and decisions that have had a significant impact on the climate change process, examining the role of climate change communication in the political discourse of sustainable development policies [56,57]. 

### 5.3. Analysis of Research Frontiers

**Figure 8:**
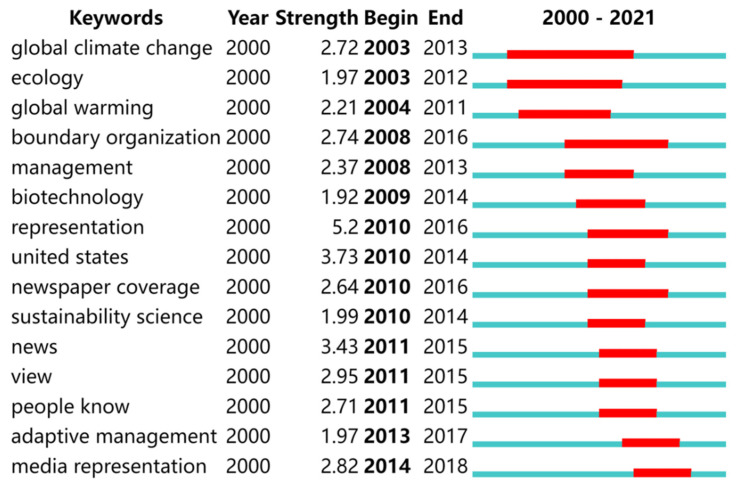
Top 15 keywords with the strongest citation bursts.

CiteSpace can detect the sudden emergence or fading of literature keywords in the research field within a period of time through the bibliometric statistical calculation method, and this detection method can visually present the frontier knowledge hotspots and their evolutionary trends in the field. By identifying and tracking the emergent keywords, we can better understand the evolution of research hotspots over time and judge the development trend at the same time, from which we can predict the scientific problems that may emerge in the future which need to be solved urgently, and then provide the information as a reference for the future direction of efforts. By using the burst detection function of CiteSpace, the obtained literature was statistically analyzed, and the burst graphs of a total of 15 keywords were obtained by sorting them by the burst year and excluding the keywords that grew or faded rapidly within a short period of time (Figure 8). The red part represents the keywords that are emergent in a certain year or years, and the blue represents the keywords that are non-emergent in that time region.

The keyword statistics show that the top 5 keywords in climate change communication research between 2000 and 2021 are “representation”, “United States”, “news”, “view” and “media representation”. The main research hotspots in climate communication can be divided into three phases according to the duration of the heat of the emergent words.

The first phase, from 2003–2013, focused on responses relating to natural climate change rather than climate communication, how news coverage of climate change influenced public perceptions of uncertainty in scientific findings [58], and the impact of global environmental assessments on developed and developing countries [59,60]. 

In the second phase (from 2008–2016), climate communication practices and strategies received more attention, and a variety of communication tools such as narratives and rhetoric of games and climate communication public works were included in the study [61,62]. Beyond the public, researchers began to focus on broader climate communication practices, such as how early risk communication and consensus should be reached between scientists, planners, and policymakers [63,64]. 

In the third phase, 2011–2018, people’s perceptions and climate-resilient behavior became the focus of research, and American scholar Oregon proposed a dialogic communication model of communication that would enable participants to gain a deeper understanding of knowledge, beliefs, perceptions, values, and barriers relating to climate communication [65]. 

Media presentation has emergent words at different stages, which we believe is largely attributable to the iterations of media technology and related to the important role that the media has assumed in public-facing climate communication. Newspapers, television, news, and social media became the arenas for studying climate communication practices [66,67,68,69]. In general, the early areas of climate communication research were more focused, and after 2004, the areas of research have become more dispersed and diverse.

## 6. Discussion

This paper takes the research literature on climate change science communication published in the WOS core database from 2000–2021 as the object of study, uses CiteSpace to systematically analyze the development history of international climate communication research, and summarizes the research findings through data analysis as follows:

(1) For the general trend of research, the field of climate communication research has shown rapid development, with increasing research heat and expansion of influence. The relevant literature was relatively small before 2007, with no more than five annual publications, and it entered a phase of rapid growth from 2008, with more than 100 research articles published for the first time in 2018. The change in the volume of publications is closely related to the fact that human actions continue to influence and accelerate the rate of change in the Earth’s environment and climate, and that natural events (such as the increasing frequency of extreme weather) and social events (international climate conferences and national climate frameworks, etc.) have attracted the attention of scholars, all of which have driven further research on climate communication. 

(2) In terms of the spatial distribution of literature output, researchers in developed countries are the main population engaged in climate communication research and have accumulated rich academic results in this field, while developing countries, represented by China and South Africa, are actively involved in research on this topic. 

(3) Research on climate change in general encompasses natural science understanding of climate change, policy research on climate change, and research on the communication of climate change in science.In recent years, public perception, adaptive management and media presentation have been hot topics of research in the field of climate communication. 

(4) Last but not least, research in this field is dominated by individual research and collaboration within small groups. Cooperation and collision between different disciplines, institutions and even countries are likely to produce more quality research.

## 7. Conclusions

The overall study shows that climate communication is showing strong momentum in the present and future..The number of publications in the first four months of 2021 already surpassed the number of publications for the whole year of 2020 and the average for the third period (2014–2021). We believe that this research area is currently showing great potential and scope. 

The extent to which developing countries influence the global climate change process is not proportional to their ability to resist global climate change, and since there are huge differences between developing and developed countries in multiple latitudes such as economic development, energy structure, climate characteristics, and the level of scientific literacy of citizens, under the framework of the United Nations Framework Convention on Climate Change, researchers in developing countries should pay more attention and effort to the theory and practice research in climate change communication.

While the global pandemic and the war may have opened up interesting analogs for this field, with the emergence of new climate policies, new national agreements, and new communication technologies, policy implications, effective case studies, and the impact of electronic and virtual media on public perceptions, attitudes, and behaviors are likely to be new research topics.

## Figures and Tables

**Table 1:** Top 20 countries in the number of articles published.

No.	Country/Region	Year of First Appearance	Number of Articles Issued
1	USA	2000	489
2	England	2008	163
3	Germany	2008	115
4	Australia	2009	103
5	Canada	2008	93
6	Sweden	2008	57
7	Netherlands	2009	54
8	Switzerland	2008	47
9	Spain	2009	47
10	China	2010	46
11	France	2009	40
12	Italy	2009	31
13	Norway	2013	31
14	New Zealand	2009	30
15	Portugal	2009	27
16	Austria	2011	26
17	Finland	2010	25
18	Scotland	2010	25
19	Wales	2009	24
20	South Africa	2009	22

**Table 2:** The most prolific authors in the field of climate communication science research.

No.	Author	Year of First Appearance	Number of Articles Issued
1	Anthony Leiserowitz	2013	9
2	Edward Maibach	2014	8
3	Brigitte Nerlich	2009	8
4	Rusi Jaspal	2013	5
5	Lauren Feldman	2014	4
6	Nelya Koteyko	2010	4
7	Andrea M Feldpausch-parker	2013	4
8	Mike S Schafer	2018	4
9	P Sol Hart	2015	4
10	Ann Bostrom	2013	3
11	Adam Corner	2009	3
12	Dominique Brossard	2018	3
13	Baruch Fischhoff	2011	3
14	Abel Gustafson	2018	3
15	Alistair J Hobday	2014	3
16	Bruno Takahashi	2011	3

**Table 3:** Top 20 research institutions with the highest number of published papers.

No.	Name of Institution	Year of First Appearance	Number of Articles Issued
1	George Mason Univ	2009	27
2	Cornell Univ	2008	22
3	Cardiff Univ	2009	20
4	Univ Wisconsin	2004	18
5	Univ Oxford	2007	17
6	Chinese Acad Sci	2015	17
7	Univ British Columbia	2005	16
8	Univ Michigan	2004	15
9	Yale Univ	2014	13
10	Univ Washington	2008	13
11	Carnegie Mellon Univ	2002	13
12	Colorado State Univ	2009	12
13	Univ Lisbon	2015	11
14	Lund Univ	2002	11
15	Arizona State Univ	2004	11
16	Univ Leeds	2015	10
17	Univ Helsinki	2014	10
18	Univ Exeter	2014	10
19	Univ Arizona	2016	10
20	Australian Natl Univ	2002	10

**Table 4:** Statistics of the Top 15 keywords.

Number	Key Words	Frequency	Year of First Appearance
1	climate change	645	2000
2	science	424	2005
3	communication	213	2005
4	knowledge	157	2004
5	science communication	145	2009
6	policy	118	2005
7	impact	104	2003
8	perception	103	2003
9	risk	99	2003
10	management	97	2002
11	information	89	2005
12	uncertainty	72	2003
13	adaptation	72	2003
14	media	66	2005
15	attitude	61	2006

## Data Availability

The experiment data used to support the findings of this study are included in the article.
